# Collagen Hydrolysate from the Scales of Mozambique Tilapia (*Oreochromis mossambicus*) Improve Hair and Skin Health by Alleviating Oxidative Stress and Inflammation and Promoting Hair Growth and Extracellular Matrix Factors

**DOI:** 10.3390/md21090475

**Published:** 2023-08-29

**Authors:** Su Bin Hwang, Hyeon Ju Park, Bog-Hieu Lee

**Affiliations:** Department of Food and Nutrition, Chung-Ang University, Anseong 17546, Republic of Korea; subin_0719@nate.com (S.B.H.); h_ju96@naver.com (H.J.P.)

**Keywords:** collagen hydrolysate, hair growth, antioxidant, anti-inflammation

## Abstract

Fish-derived collagen hydrolysate (CH) has shown promise in improving hair and skin health. Therefore, this study sought to comprehensively assess the effects of CH extracted from Mozambique tilapia (*Oreochromis mossambicus*) scales on hair and skin using in vitro and in vivo models. Human dermal papilla cells (hDPCs) were used for antioxidant and gene expression analyses, while C57BL/6 mice were orally administered CH for six weeks to assess hair growth patterns. The mice were divided into four groups: negative control (NC; distilled water), positive control (PC; 1 mg/kg finasteride), CH500 (500 mg/kg BW CH), and CH1000 (1000 mg/kg BW CH). CH mitigated catalase activity reduction in hDPCs, increased IGF-1 and VEGF levels, and decreased TGF-β1, TNF-α, and IL-1β expression. In vivo, CH treatment improved hair growth index, length, diameter, weight, and density. Scanning electron microscopy revealed reduced hair damage. Moreover, CH up-regulated IGF-1, VEGF, Elastin, and HAS2 mRNA expression while down-regulating TNF-α and IL-1β. CH enhanced hair shine, growth, and skin health while alleviating inflammation. These findings demonstrate the potential of CH in alleviating oxidative stress, promoting hair growth, and enhancing skin health, both in vitro and in vivo. Fish-derived CH offers a cost-effective and bioavailable option for improving hair and skin health.

## 1. Introduction

Hair serves various functions in mammals, including physical protection, dispersion of sweat and sebum, sensory and tactile functions, and social interaction. In human society, hair holds significant psychosocial importance, and unhealthy hair and hair loss can cause considerable mental stress [1]. The causes of hair loss are predominantly genetic, nutritional, and environmental factors, primarily linked to oxidative stress caused by oxygen free radicals, which can be excessively generated due to various factors such as aging, fatigue, ultraviolet rays, chemicals, and stress [2]. Oxidative stress causes damage to intracellular molecules and plays a crucial role in several diseases, including skin diseases, and has been associated with skin and hair aging [3]. Oxidative stress can induce hair loss by triggering apoptosis and inflammation in hair follicles and skin and delaying the anagen phase of the hair cycle, thus affecting hair shine, diameter, and the integrity of the extracellular matrix, therefore interrupting healthy hair growth [4]. Therefore, antioxidant enzymes such as superoxide dismutase (SOD), catalase (CAT), and glutathione peroxidase (GSH-Px) are crucial for preventing hair loss by counteracting oxidative stress [3]. CAT, in particular, is an important antioxidant enzyme that protects tissues by converting hydrogen peroxide into water and oxygen, therefore limiting ROS production [5,6,7]. Moreover, the up-regulation of pro-inflammatory cytokines around hair follicles is known to contribute to hair loss by promoting ROS production and initiating early degradation and apoptosis of hair follicles. Up-regulation of pro-inflammatory cytokines such as tumor necrosis factor-α (TNF-α) and interleukin 1 (IL-1) is linked to hair loss, as they induce apoptosis and inhibit hair follicle proliferation. In particular, studies have shown a significant positive correlation between TNF-α levels and hair loss [8]. IL-1 also plays a significant role in hair loss by directly inhibiting hair follicles through the regulation of the immune/inflammatory system [9]. In vitro studies have demonstrated that IL-1α, IL-1β, and TNF-α cause toxicity, reduce the size of stromal cells in the follicle bulb, degrade hair follicle melanocytes, and lead to abnormal differentiation and keratinization [8,9]. Therefore, the regulation of pro-inflammatory cytokines holds potential as a crucial marker for the treatment and prevention of hair loss. Therefore, to prevent hair loss, it is crucial to protect from the oxidative stress and alleviate inflammation by regulating antioxidant enzymes and inflammation-related cytokines [3,9,10].

Oxidative stress and inflammatory cytokines are fundamental factors that inhibit hair growth and directly affect the hair cell cycle. Mature and healthy hair is composed of hair shafts as the basic units [11,12,13], which grow through three phases of the hair cycle: anagen, catagen, and telogen. Hair shafts are formed and shed in each phase, and weaker hair shafts increase the risk of hair loss. Therefore, hair shaft formation and activation play a vital role in hair growth [14]. In response to epithelial-mesenchymal signals, epithelial cells grow downward, forming the outer root sheath (ORS), inner root sheath (IRS), and hair shaft through proliferation and differentiation [14,15]. Regulating hair growth-promoting factors in the hair bulb is essential for hair shaft formation [16]. Insulin-like growth factor 1 (IGF-1), a well-known hair growth-promoting factor, controls the hair growth phase and hair shaft differentiation, in addition to preventing anagen-to-catagen transition [17]. Additionally, vascular endothelial growth factor (VEGF), which is widely recognized as a hair growth-promoting factor, influences hair shaft differentiation in the hair growth phase by promoting the proliferation and differentiation of hair root cells for hair shaft formation [18]. Transforming growth factor-beta 1 (TGF-β1) inhibits hair shaft formation and development by inducing apoptosis, increasing telogen hair follicles, and promoting the transition of growing hair follicles into catagen hair follicles [19,20,21,22,23].

Interestingly, skin and hair health are closely intertwined. The skin comprises the epidermis, dermis, and subcutaneous tissue. The dermal papilla (DP) and dermal sheath (DS) originate from the same cellular progenitors as interfollicular dermal fibroblasts (DFs). Interfollicular fibroblasts are responsible for synthesizing and maintaining the extracellular matrix (ECM) of the dermis, whereas the DP and DS play crucial roles in regulating hair growth [24]. Collagen, elastin, and hyaluronic acid, which are components of the dermal ECM, maintain skin health by preserving its structure and elasticity. The skin becomes thinner when its integrity is compromised, thus affecting its wound healing and regenerative abilities [25,26]. The aging process affects not only the surface of the skin but also skin appendages, such as hair follicles [27]. The regeneration of hair follicles relies on epithelial-mesenchymal interactions between stem/progenitor cells and fibroblasts, including dermal papilla cells (specialized fibroblasts of the hair follicle) [28,29]. The interactions between follicular keratinocytes and dermal papilla cells govern hair formation and cycling [30,31]. Therefore, maintaining healthy skin, including ECM production or remodeling, is crucial for hair health.

Currently, only topical minoxidil and oral finasteride have been approved by the US Food and Drug Administration (US FDA) for the treatment of hair loss. Patients suffering from hair loss often complain about scalp pruritus, scalp scaling, scalp irritation, undesirable hair texture, and the need to apply medication twice a day. In addition, finasteride has several side effects such as anxiety, depression, erectile dysfunction, loss of libido, and reduced ejaculatory volume [32]. However, research on hair growth therapies has mostly focused on direct topical treatments, whereas oral treatments have remained largely unexplored [33].

The intake of collagen hydrolysate (CH) has been found to play various roles in the body, including bones, Achilles tendon, and skin, and has recently been recognized for promoting hair and skin health [34,35,36,37,38,39,40]. Specifically, in comparison with mammalian-based collagen, CH derived from fish has advantages such as high collagen content, environmental friendliness, low inflammatory response, and toxicity, no risk of transmitting diseases, and biocompatibility, and enhanced absorption by the human body due to its low molecular weight, suggesting that the low molecular collagen from fish CH can be greater at improving bioavailability and skin health [41,42,43,44].

Fish-isolated low-molecular-weight bioactive peptide is a popular safe cosmetic biomaterial considered to be a good source of antioxidant and anti-inflammatory activities [45,46,47,48]. The antioxidant properties of hydrolyzed collagen are conditioned to the size of the molecule: the lower the molecular weight of peptides, the greater the ability to donate an electron or hydrogen to stabilized radicals [35,49,50]. In previous studies, oral administration of low-weight-molecular collagen peptides (approximately 800 Da) derived from chicken feet can be detected in the skin until 14 days in rats, confirming the absorption and effectiveness of oral administration [51]. In rats and mice, absorbed collagen hydrolysates (Gly-Pro-Hyp) remain in the plasma for several hours, with a peak concentration of ~2–4 h after administration, and can be detected in various tissues, especially in the skin [51,52,53]. Marine-derived collagen peptides (CPs) were derived from fish skin (e.g., *Pollachius virens*, *Hippoglossus hippoglossus*, and *Pleuronectes platessa*) easily penetrate the gastrointestinal wall through blood circulation and are mainly deposited in the skin [54]. A higher level of Gly-Pro-Hyp in plasma was detected after the oral intake of low-molecular-weight collagen hydrolysates or collagen tripeptide compared to high molecular weight collagen peptide. In addition, Gly-Pro-Hyp and Pro-Hyp were stable in gastrointestinal fluid and plasma without being decomposed by gastric acid and enzymes, pancreatin, or plasma peptidases [55]. These results indicate that the oral intake of low-molecular-weight CPs can be an efficient approach to taking bioactive peptides owing to the enzymatic stability and intestinal permeability of low-molecular-weight peptides [55,56]. Moreover, oral administration of low-molecular-weight peptides improves skin properties such as elasticity, skin moisture, and trans-epidermal water loss [35]. Gly-Pro-Val-Gly-Pro-Ser peptides from *Oreochromis niloticus* improved skin dryness and wrinkle formation against UV-B-induced skin photoaging through antioxidant and anti-inflammatory properties [57]. Additionally, among fish CH, CH derived from the scales of Mozambique tilapia (*Oreochromis mossambicus*) has been found to promote wound healing and cell proliferation [58,59].

Additionally, in a previous study, we demonstrated that oral administration of CH promotes dermal papilla cell proliferation and hair growth [60]. Recently, research on marine-derived collagen hydrolysate has been increasing. Moreover, fish-derived CH can be taken orally, thus providing a more convenient route of administration compared to topical treatment. However, previous studies have not confirmed whether the CH derived from the scales of Mozambique tilapia (*Oreochromis mossambicus*) has positive effects on clinical hair characteristics as well as antioxidant and anti-inflammatory properties. Therefore, our study sought to confirm the effects of oral CH intake on skin and hair health including clinical hair characteristics such as hair shine, diameter, and elasticity in depilation-induced mice.

## 2. Results

### 2.1. Antioxidant Effects of CH on H_2_O_2_-Induced hDPCs

**Figure 1:**
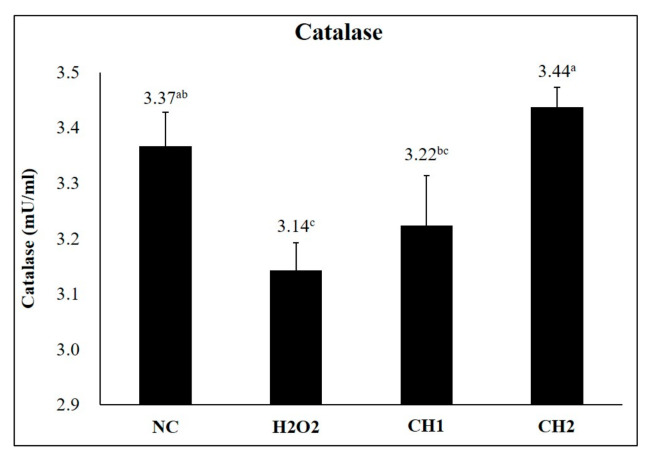
Effect of CH on catalase activity. Values with different superscript letters are significantly different at *p* < 0.05. The results represent the mean ± SE. The hDPCs were seeded at a density of 3 × 10^4^ cells/plate. Once the cell plates reached 70% confluence, they were treated with H_2_O_2_ for 24 h. After 24 h, each treatment was treated for an additional 24 h at 5% CO_2_ and 37 °C. NC: negative control (DMEM); H_2_O_2_: H_2_O_2_ (750 μM); CH1: collagen hydrolysate 62.5 μg/mL; CH2: collagen hydrolysate 125 μg/mL.

The H_2_O_2_ group exhibited a significant decrease in catalase activity compared to the NC group (*p* < 0.05). However, CH2 (125 μg/mL) and CH1 (62.5 μg/mL) restored catalase activity to levels similar to those of the NC group (*p* < 0.05) (Figure 1).

### 2.2. Effects of CH on Cytokine mRNA Expression of LPS-Induced hDPCs

**Figure 2:**
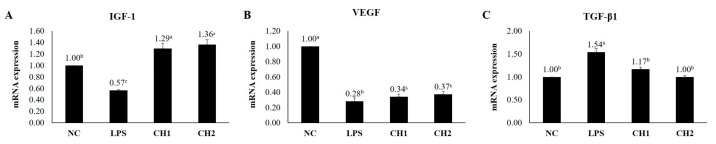
Results of RT-qPCR. Effect of collagen hydrolysate on hair-related cytokines. (**A**) IGF-1, (**B**) VEGF, and (**C**) TGF-β1. Values with different superscript letters are significantly different at *p* < 0.05. The results are the mean ± SE. The hDPCs were seeded at a density of 3 × 10^4^ cells/plate. Once the cell plates reached 70% confluence, they were treated with LPS for 24 h. After 24 h, each treatment was treated for an additional 24 h at 5% CO_2_ and 37 °C. NC: negative control (DMEM); LPS: LPS (400 ng/mL); CH1: collagen hydrolysate 62.5 μg/mL; CH2: collagen hydrolysate 125 μg/mL.

**Figure 3:**
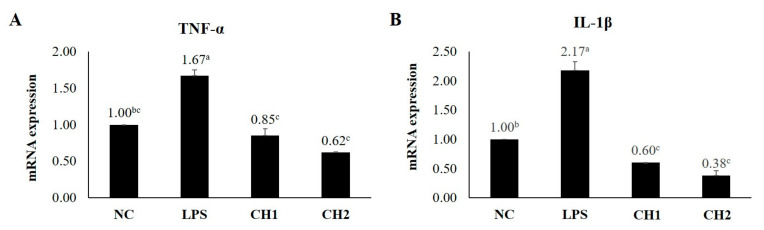
Results of RT-qPCR. Effect of collagen hydrolysate on inflammatory cytokines. (**A**) TNF-α and (**B**) IL-1β. Values with different superscript letters differ significantly at *p* < 0.05. The results represent the mean ± SE. The hDPCs were seeded at a density of 3 × 10^4^ cells/plate. Once the cell plates reached 70% confluence, they were treated with LPS for 24 h. After 24 h, each treatment was treated for an additional 24 h at 5% CO_2_ and 37 °C. NC: negative control (DMEM); LPS: LPS (400 ng/mL); CH1: collagen hydrolysate 62.5 μg/mL; CH2: collagen hydrolysate 125 μg/mL.

To assess the effects of CH treatment on LPS-induced hDPCs, the mRNA expression levels of hair growth-promoting/inhibitory factors (VEGF, IGF-1, and TGF-β1) and inflammatory factors (TNF-α and IL-1β) were quantified using RT-qPCR (Figure 2 and Figure 3). The results were normalized to the NC group. Both CH groups exhibited increased mRNA expression of IGF-1 and VEGF and decreased mRNA expression of TGF-β1 compared to the PC group. The CH2 group exhibited the highest mRNA expression level of IGF-1, which promotes the proliferation of hair follicle epithelial cells and inhibits the transition from the anagen phase to the telogen phase, followed by the CH1, NC, and PC groups (*p* < 0.05). VEGF, a factor that stimulates hair root cell differentiation and enhances hair follicle size and thickness through improved blood circulation, did not exhibit significant differences between the CH groups and the PC group. TGF-β1 mRNA expression, which is responsible for inducing hair follicles from the anagen phase to the telogen phase by promoting hair follicle apoptosis, was highest in the PC group. The CH groups exhibited reduced TGF-β1 expression compared to the PC group (*p* < 0.05). Regarding inflammatory cytokines, TNF-α and IL-1β are well-known major factors. The CH groups exhibited lower expression levels of these inflammatory factors compared to the PC group. Specifically, the CH groups exhibited lower mRNA expression levels compared to the NC group (*p* < 0.05).

### 2.3. Visual Observation of Hair Growth after CH Oral Administration

**Figure 4:**
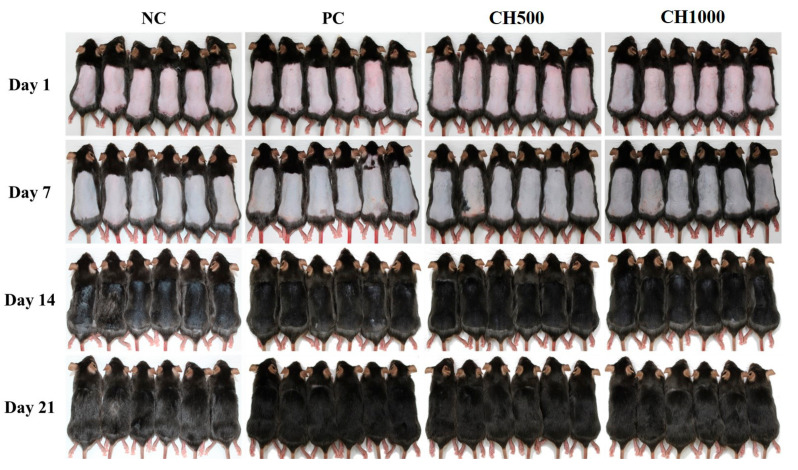
Effects of CH on the dorsal skin of C57BL/6 mice following depilation. The mice were orally administered with distilled water (vehicle), finasteride (1 mg/kg in distilled water), and CH (500 and 1000 mg/kg in distilled water) before and after depilation for a duration of 6 weeks. Dorsal skin photographs were taken on days 1, 7, 14, and 21 post-depilation. NC: negative control (distilled water); PC: positive control (finasteride); CH500: CH 500 mg/kg; CH1000: CH 1000 mg/kg.

To assess the hair growth pattern in mice following CH oral intake, photographs were taken at 7-day intervals over a period of 21 days (Figure 4). On the first day of hair removal, all groups exhibited pink dorsal skin color. After 7 days, the dorsal skin color of all groups transitioned from pink to gray, with the CH1000 group appearing the darkest shade of gray. By day 14, hair growth became visually evident in all groups, progressing to the point where the skin was no longer visible in the PC, CH500, and CH1000 groups, unlike the NC group. However, in the NC group, compared to the other groups, partial visibility of the dorsal skin and weaker hair growth were observed. By the 21st day, complete hair growth was observed in all groups, with a notable decrease in hair shine, particularly in the NC group compared to the other groups. The CH groups (CH500, CH1000) displayed a glossy hair growth pattern with dark black hair, confirming that oral intake of CH promotes hair growth by inducing the anagen phase of hair.

### 2.4. Effects of CH on Hair Regrowth in Mice

**Figure 5:**
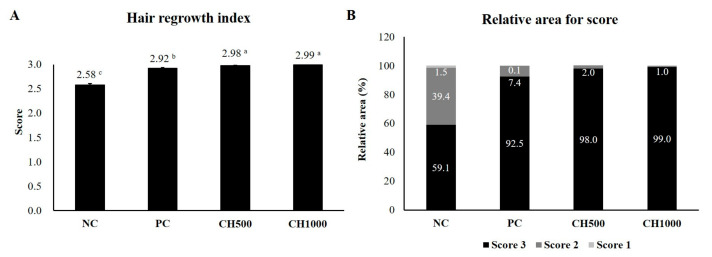
Effects of CH on hair regrowth score in depilation-induced C57BL/6 Mice. (**A**) Hair regrowth index. (**B**) Relative area for a score. The hair regrowth index was calculated based on dorsal skin photos taken on day 14. The size of the complete black area (representing regrown hair) was measured using the ImageJ software (Java 8). Values with different superscript letters were significantly different (*p* < 0.05). The results are reported as mean ± SE (*n* = 5/group). NC: negative control (distilled water); PC: positive control (finasteride); CH500: CH 500 mg/kg; CH1000: CH 1000 mg/kg.

On day 14, dorsal skin photographs of the mice were taken, and the hair regrowth score and area were calculated using the ImageJ program (Figure 5A,B). The hair regrowth score ranged from 0 to 3, with a higher score indicating more complete hair growth. The NC group had the lowest hair regrowth score at 2.58 ± 0.03 (*p* < 0.05). Afterward, the PC (2.92 ± 0.01), CH500 (2.98 ± 0.00), and CH1000 (2.99 ± 0.00) groups exhibited progressively higher hair regrowth scores. The CH group exhibited a significantly higher hair regrowth score compared to the PC group, with the CH1000 group achieving a score close to 3 points. Similarly, using the ImageJ program to measure the back area of each mouse based on the hair regrowth score, the PC and CH groups had significantly larger areas with a score of 3 compared to the other scores. In the NC group, the proportion of areas with scores of 3 points and 2 points was similar at a ratio of 1.5:1. The CH group had the highest proportion among the areas with a score of 3 points, followed by the PC and NC groups (*p* < 0.05). Conversely, in the areas with a score of 2 points, the treatments exhibited the following order: NC > PC > CH (*p* < 0.05). Areas with a score of 1 point were present in the NC group but not in the PC and CH groups. The CH group exhibited a high hair regrowth index, with most of the areas achieving a score of 3 points, indicating complete hair growth. These findings confirm that the hair growth-promoting effect of CH is comparable to that of finasteride, the positive control group.

### 2.5. Effects of CH on the Improvement of Clinical Characteristics of Hair

On day 21, mouse hair was randomly plucked to measure hair diameter, length, weight, and density per area (Table 1). Hair diameter was significantly smaller in the NC group (*p* < 0.05), but no significant difference was observed between the PC and CH groups. Similar to the hair diameter, hair length in the NC group was the shortest (*p* < 0.05), with no significant difference observed between the PC and CH groups. The NC group exhibited the lowest hair weight (0.019 ± 0.001 g), followed by PC (0.023 ± 0.001), CH500 (0.029 ± 0.001), and CH1000 (0.035 ± 0.000) (*p* < 0.05). Hair density was calculated using a hair and scalp analyzer. The NC group exhibited the lowest hair density at 48.0 ± 4.853 points (*p* < 0.05). The CH500 (61.5 ± 3.025) and CH1000 (68.8 ± 1.706) groups exhibited higher hair density compared to the PC (59.8 ± 3.782) group. The CH group tended to exhibit higher hair diameter, length, and density values, with a significant increase in hair weight compared to the positive control. These findings indicate that CH intake improved the clinical properties of hair.

### 2.6. Effect of CH on Hair Shine Enhancement Measured by SEM

**Figure 6:**
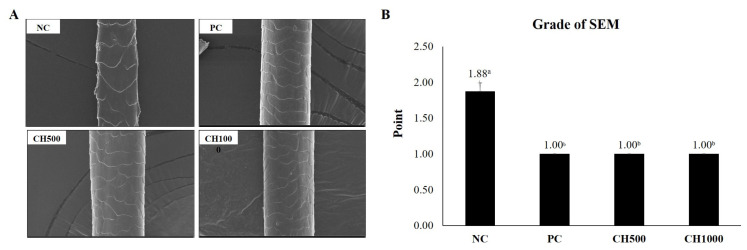
Effect of CH on hair shine. (**A**) SEM images of mouse hair and (**B**) Grade of SEM. Values with different superscript letters indicate significant differences at *p* < 0.05. The results are reported as mean ± SE. NC: negative control (distilled water); PC: positive control (finasteride); CH500: CH 500 mg/kg; CH1000: CH 1000 mg/kg.

To measure hair shine, a crucial clinical characteristic of hair, mouse hair was randomly plucked on day 21 and the surface of the hair was examined using SEM (Figure 6). The degree of improvement in hair gloss was evaluated and categorized into 5 grades based on the condition of the hair surface cuticle. In the NC group, the hair exhibited an irregular overlay, lifting, and a rough surface, resulting in a grade 2 (1.88 ± 0.12). However, in the PC group, the irregular overlay exhibited improvement compared to the NC group, and the lifted state and rough surface were noticeably alleviated, leading to a grade 1 classification (1.00 ± 0.00). Similarly, the CH group demonstrated a significantly improved irregular overlay compared to the NC group, and the rough surface was alleviated, also achieving a grade 1 (1.00 ± 0.00). These findings indicate that the consumption of CH enhances hair shine by improving the condition of the hair cuticle layer.

### 2.7. Effects of CH on Cytokine mRNA Expression of Mouse Dorsal Skin

**Figure 7:**
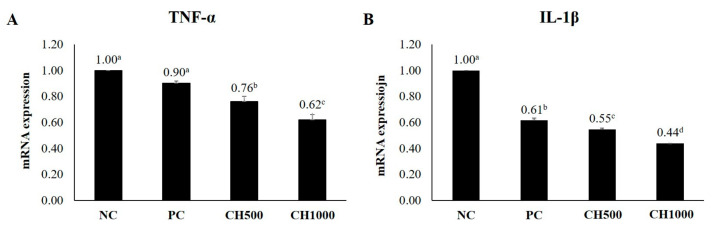
RT-qPCR results. Effect of CH on hair-related cytokines. (**A**) TNF-α and (**B**) IL-1β. Values with different superscript letters indicate significant differences at *p* < 0.05. The results are reported as mean ± SE. NC: negative control (distilled water); PC: positive control (finasteride); CH500: CH 500 mg/kg; CH1000: CH 1000 mg/kg.

**Figure 8:**
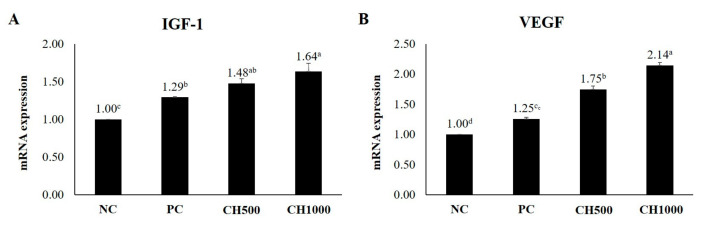
RT-qPCR results. Effect of CH on hair-related cytokines. (**A**) IGF-1 and (**B**) VEGF. Values with different superscript letters are significantly different at *p* < 0.05. The results are reported as the mean ± SE. NC: negative control (distilled water); PC: positive control (finasteride); CH500: CH 500 mg/kg; CH1000: CH 1000 mg/kg.

**Figure 9:**
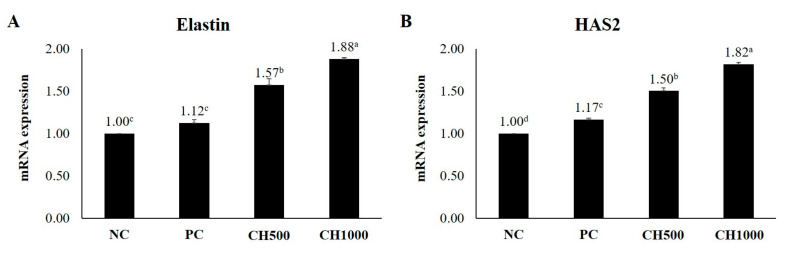
RT-qPCR results. Effect of CH on hair-related cytokines. (**A**) Elastin and (**B**) HAS2. Values with different superscript letters indicate significant differences at *p* < 0.05. The results are reported as mean ± SE. NC: negative control (distilled water); PC: positive control (finasteride); CH500: CH 500 mg/kg; CH1000: CH 1000 mg/kg.

To examine the effects of CH on mouse dorsal skin, mRNA expression levels of inflammatory factors (TNF-α/IL-1β), hair growth-promoting factors (IGF-1/VEGF), and extracellular matrix (ECM) production-related factors (Elastin/HAS2) were measured using RT-qPCR (Figure 7, Figure 8 and Figure 9). The results were normalized to the NC group. Both CH groups exhibited a significant reduction in the mRNA expression levels of TNF-α and IL-1β, in addition to an attenuation of the increased expression of IGF-1 and VEGF compared to the NC and PC groups. Furthermore, the CH groups exhibited increased Elastin and HAS2 mRNA expression compared to the PC group (*p* < 0.05).

## 3. Discussion

Our study investigated the effects of CH scales on H_2_O_2_- and LPS-exposed hDPCs in vitro. We observed that CH improved catalase activity compared to the PC group (*p* < 0.05) and promoted the expression of hair growth factors (IGF-1 and VEGF) while decreasing the expression of hair growth inhibiting factor (TGF-β1) and inflammatory factors (TNF-α and IL-1β). In vivo, we assessed the clinical characteristics of hair and skin health. CH increased hair length, diameter, weight, and density compared to the NC group. Additionally, our SEM analyses demonstrated that CH reduced mild damage and improved hair shine compared to the NC group. Furthermore, in RT-qPCR analysis, CH enhanced the expression of hair growth factors and ECM production factors while decreasing inflammatory factors (*p* < 0.05) compared to the NC group.

Enhancing the activity of these antioxidant enzymes including CAT, can delay skin and hair aging, prevent hair loss by modulating hair growth factors such as IGF-1 and VEGF, and mitigate oxidative stress-induced damage to the extracellular matrix through inhibition of oxidative stress, anti-inflammatory effects by reducing the expression of inflammatory cytokines (IL-6, IL-1β, TNF-α), and preservation of ECM integrity by increasing hyaluronic acid levels through up-regulation of HAS [2,61,62,63]. The hair shaft consists of three layers: the cuticle, cortex, and medulla [64]. The cuticle, which surrounds and protects the cortex, plays a crucial role in hair shine. Undamaged cuticles contribute to a smooth hair surface that reflects light, resulting in shiny hair [64,65,66]. The cortex, which is situated between the cuticle and medulla, determines hair elasticity and strength [64,67]. The medulla, the innermost layer of the hair, provides structural support and influences hair thickness [66]. Thus, to improve clinical characteristics such as elasticity, shine, and thickness, it is essential to enhance the cuticle, cortex, and medulla. Previous studies have shown that probiotic consumption in aged male animals increased subcuticular folliculogenesis, resulting in luxuriant fur and improved anti-inflammation [68]. Additionally, IGF-1 and EGF have been found to jointly stimulate hair shaft elongation, promote the transition of the hair cycle, and enhance cell proliferation in the outer root sheaths and dermal papilla [69]. Platelet-rich plasma treatment has also demonstrated positive effects on hair diameter and density by up-regulating IGF-1 and VEGF, which influence hair growth and angiogenesis [70]. In previous studies, we have also demonstrated the potential of the oral administration of fish-derived collagen from the scales of Mozambique tilapia (*Oreochromis mossambicus*) exhibited a high A/T ratio, which is the marker for hair follicle growth in dorsal of mice skin via hematoxylin and eosin staining. Also, CH significantly increased the expression of hair growth factors (IGF-1, VEGF, krt27, Gprc5d, and Ki67) and decreased the growth inhibitory factor (TGF-β1), up-regulating the Wnt/β-catenin pathways and down-regulating the BMP pathways [60]. An oral supplement of bioactive fish-derived collagen hydrolysates/peptides or complex improved hair growth, decreased hair shedding, and increased hair thickness with good tolerability on the subjects with alopecia or chronic telogen effluvium in several clinical trials [36,71,72].

Marine-derived L-fucose and chondroitin sulfate disaccharide have been shown to support skin and hair health by promoting the proliferation of dermal fibroblasts and dermal papilla cells, increasing ECM molecule production (collagen and elastin), and supporting the anagen phase of the hair cycle (anagen phase) [73]. Collagen VI is an extracellular matrix molecule that is abundantly expressed in the skin. Injection of purified collagen VI alleviated the abnormal wound-induced hair regrowth in Col6a1-/- mice [74]. Pro-Hyp in keratinocytes in mouse skin exhibited an up-regulation of hair cycle-related genes such as Gprc5d and Krt27 [75]. Hydroxyproline-containing peptides have been shown to improve skin conditions by alleviating oxidative stress, inhibiting inflammation, regulating cell behavior, and maintaining the extracellular matrix (ECM) [76]. CPs are rich in proline-hydroxyproline (Pro-Hyp) dipeptide and promote cell proliferation and hyaluronic acid synthesis in human dermal fibroblasts [77]. In hairless mice, oral administration of low-molecular-weight CH derived from fish collagen hydrolysate promotes the recovery of collagen fibers and elastic fibers in skin damaged by UVB irradiation [56]. Application of type II collagen from cartilage from *Acipenser baerii* substantially increased the expression of growth factors, mostly in the epidermis (rich in keratinocytes) and partially in the dermis (rich in fibroblasts). Collagens, in interaction with other ECM components, such as elastin, laminin, fibronectin, proteoglycans, and glycosaminoglycans, form fibrous networks that influence cell growth, attachment, migration, and differentiation [78]. Moreover, adequate moisturization is vital for maintaining healthy skin, which depends on the level of hyaluronic acid in the stratum corneum and the activity of hyaluronan synthase (HAS) which maintains the concentration of hyaluronic acid in the skin. Oral ingestion of collagen tripeptides (1000 mg) from the skin of Nile tilapia (*Oreochromis niloticus*) improved skin hydration in women aged 40–60 compared to the placebo group [44]. Oral intake of fish-derived CPs (500 and 1000 mg/kg BW) increased skin hydration and decreased wrinkle formation, up-regulating the mRNA and protein of HAS and skin-hydrating factors (filaggrin and involucrin) compared to the UVB-irradiated group [40].

Recently, Gly-Pro-Val-Gly-Pro-Ser peptide from fish scales of Nile tilapia (*Oreochromis niloticus*), which is similar to the indicator in our sample was reported to possess antioxidant activity by increasing SOD, CAT, and GPx activity in addition to exhibiting anti-inflammatory effects by reducing the expression of inflammatory cytokines (IL-6, IL-1β, and TNF-α), and preserving ECM integrity by increasing hyaluronic acid levels through up-regulation of HAS in ultraviolet-B (UVB) irradiated in vitro and in vivo, suggesting that the peptide can be effective against UVB induced skin photoaging by improving skin dryness and wrinkle formation through antioxidant and anti-inflammatory properties [57]. These findings suggest that the clinical properties of hair and skin health are influenced by antioxidant, anti-inflammatory, hair growth, and ECM production properties. However, we have some limitations in this study. This is the first comprehensive study of collagen hydrolysate from the fish scales oral intake on skin and hair health. Therefore, further study is needed to analyze and characterize the components of the sample and confirm various antioxidant properties including SOD, GPx, direct ROS, etc., and molecular analysis including immunology and signaling pathways in-depth.

## 4. Materials and Methods

### 4.1. Cell Culture and Materials

The human dermal papilla cells (hDPCs) used in this study were obtained from Cell Engineering for Origin (CEFO BIO, Seoul, Republic of Korea). Dulbecco’s modified Eagle’s medium (DMEM) supplemented with 1% antibiotics and 10% fetal bovine serum (FBS) was used for cell culture. The cells were incubated at 37 °C in a humidified atmosphere with 5% CO_2._ LPS (400 ng/mL) was purchased from Sigma (L2654) and used to induce inflammation. The sample used in the study was a total fish-derived collagen protein hydrolysate extracted from the scale of Mozambique tilapia (*Oreochromis mossambicus*). To obtain collagen hydrolysate, first, tilapia scales are acid-treated to extract gelatin, decomposed by protease, and added citric acid, finally, the hydrolysates are spray-dried. The sample contained 128.8 mg/g of hydroxyproline, which is most abundant in collagen. The chemical composition of the nutrient component is 383 kcal/100 g for energy, 94% protein, 5% water content, and no ash. The sample used was individually approved raw materials by the Ministry of Food and Drug Safety in South Korea (certification number (No. 2019-12)), manufactured by Nitta Gelatin India Limited Co., (Cochin, India), and obtained from Ju Yeong NS (Seoul, Korea). For quality control purposes, the licensed indicator component was set as a peptide composed of 6 amino acids (Gly-Pro-Val-Gly-Pro-Ser), and the average content in the final raw material was 0.83 mg/g (molecular weight, 512 daltons). The sample was stored at room temperature before the experiments. The concentrations of CH used in the experiment, 62.5 μg/mL and 125 μg/mL in distilled water, were determined based on a previous study that showed the highest cell viability in hDPCs within a range of 0–1000 μg/mL [60].

To confirm the catalase activity, the hDPCs were seeded at a density of 3 × 10^4^ cells/plate. Once the cell plates reached 70% confluence, they were treated with H_2_O_2_ for 24 h to induce oxidative stress. The experimental groups included a negative control (DMEM), a positive control [H_2_O_2_ (750 μM) in DMEM], CH1 (62.5 μg/mL CH), and CH2 (125 μg/mL CH). After the induction of oxidative stress, the plates were incubated with the respective treatments for an additional 24 h at 5% CO_2_ and 37 °C. Subsequently, the cells were collected for catalase activity assay using the colorimetric method (BIOMAX, Guri, Korea).

To confirm the anti-inflammatory properties, the hDPCs were seeded at a density of 3 × 10^4^ cells/plate. Once the cell plates reached 70% confluence, they were treated with LPS for 24 h to induce inflammation. The experimental groups included a negative control (DMEM), a positive control [LPS (400 ng/mL) in DMEM], CH1 (62.5 μg/mL CH), and CH2 (125 μg/mL CH). After the induction of inflammation, the plates were incubated with the respective treatments for an additional 24 h at 5% CO_2_ and 37 °C. Subsequently, the cells were collected for RT-qPCR analysis.

### 4.2. Experimental Animals

**Figure 10:**
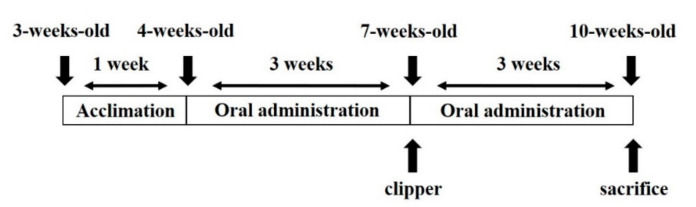
Timeline of experimental treatments and sample collection.

Three-week-old male C57BL/6 mice were purchased from Dae-Han Biolink Co. (Eumsung, Chungbuk, Korea) and acclimated to laboratory conditions for one week. The mice were housed at a temperature of 22 ± 1 °C, relative humidity of 50 ± 5%, and a 12-h light-dark cycle. The mice had ad libitum access to pelleted food and water during the acclimation period. After one week of acclimation, the mice were randomly divided into four experimental groups with eight mice per group. The experiment was conducted for a duration of six weeks (Figure 10). The experimental groups consisted of negative control (NC, distilled water), positive control (PC, 1 mg/kg finasteride), CH500 (500 mg/kg body weight (BW) CH in distilled water), and CH1000 (1000 mg/kg BW CH in distilled water). In previous hair loss studies using finasteride as reference drugs, 1 mg/kg finasteride was administered orally to mice [79,80] In addition, in the case of orally administered drugs of alopecia, to confirm the ability to inhibit 5α-reductase and block DHT formation in vivo, experiments are conducted based on oral administration of finasteride 1 mg/kg/daily when performing animal pharmacology studies [81]. Therefore, in this study as well, it is reasonable to administer finasteride orally at 1 mg/kg as a reference drug. For the choice of the doses of CH, considering that the recommended daily intake of CH is 3270 mg/day, the CH concentration was substituted into the concentration conversion formula (Human equivalent dose = Mouse dose × [Animal Km/Human Km]) [82]. Therefore, assuming that an adult weighs 40 kg to 100 kg, the concentration applied to mice is 403 mg/kg to 1008 mg/kg. Previous studies [39,40,60] on oral administration of low-molecular-weight collagen also took 500 mg/kg and 1000 mg/kg as experimental groups, so it is considered reasonable to administer oral administration at two concentrations, 500 mg/kg BW and 1000 mg/kg BW, in this study as well.

The dorsal skin of the seven-week-old mice was shaved using an animal clipper. Each group of mice received daily oral administration of 200 μL of distilled water, finasteride, or CH solution for the duration of the six-week experiment. At the end of the experiment, the mice were anesthetized with 1.2% avertin administered intraperitoneally, and the dorsal skins were obtained. All animal experimental procedures were conducted in accordance with the Guidelines for the Care and Use of Laboratory Animals of the National Institutes of Health and the guidelines established by the Animal Welfare Act. The study was approved by the Institutional Animal Care and Use Committee (IACUC) of Chung-Ang University (approval ID: A2022066). There were no significant differences in body weight and food intake among the experimental groups (*p* < 0.05), and no other specific clinical symptoms, including death, were observed during the experimental period.

### 4.3. Hair Regrowth Score and Relative Area for Each Score

To assess the impact of CH intake on hair regrowth, dorsal skin photographs were captured using a digital camera on day 14 after hair removal. The hair regrowth scores and relative areas were measured using the ImageJ software (Broken Symmetry Software, Bethesda, USA) [83]. Hair regrowth scores were assigned on a scale of 0 to 3 based on the difference in dorsal skin color. A score of 0 indicated no hair growth (pink skin), a score of 1 indicated initial hair growth (gray skin), a score of 2 indicated advanced hair growth (dark gray), and a score of 3 indicated fully grown hair (black) (Table 2). The hair regrowth score was calculated by determining the ratio of the regrown hair area to the total area, and the average score was obtained by multiplying the area ratio by the respective score for each group [84]. Therefore, the hair regrowth score ranged from 0 (minimum) to 3 (maximum).

$$\begin{array}{c} \mathrm{Hair}\;\mathrm{regrowth}\;\mathrm{score}\\ =\frac{\left[\left(\mathrm{area}\;\mathrm{of}\;\mathrm{pink}\right)\;\times \;0\;+\;\left(\mathrm{area}\;\mathrm{of}\;\mathrm{gray}\right)\;\times \;1\;+\;\left(\mathrm{area}\;\mathrm{of}\;\mathrm{dark}\;\mathrm{gray}\right)\;\times \;2\;+\;\left(\mathrm{area}\;\mathrm{of}\;\mathrm{blnak}\right)\;\times \;3\right]}{\mathrm{total}\;\mathrm{area}} \end{array}$$

### 4.4. Hair Diameter, Length, and Weight [85]

To evaluate the effect of CH ingestion on improving the clinical characteristics of mouse hair, measurements were taken for hair diameter, length, and weight. The experiment was conducted after allowing the plucked hair to acclimate at 22 ± 1 °C and 50 ± 5% humidity for approximately 2 h. Five hairs were randomly selected and microscopic images were captured at 400× magnification. Hair diameter and length were measured using the ImageJ software (Broken Symmetry Software, Bethesda, USA) with a 2× magnification. For hair weight determination, dorsal skin tissue samples were collected from the mice on day 21 under the same conditions as mentioned earlier. Hair weight was measured by weighing half of the dorsal skin tissue sample containing the hair and subtracting the weight of the corresponding dorsal skin tissue sample without hair.

### 4.5. Hair Density per Area [86]

Total hair numbers per 1 cm^2^ at day 21 were measured to determine the variations in hair density in response to CH intake. On day 21, the back of the mouse was divided into 4 areas, and the hair density was measured by capturing images using a DermoSmart+ Harris hair and scalp analyzer (Chowis Co, Yongin, Korea).

### 4.6. Scanning Electron Microscope (SEM) [87]

**Figure 11:**
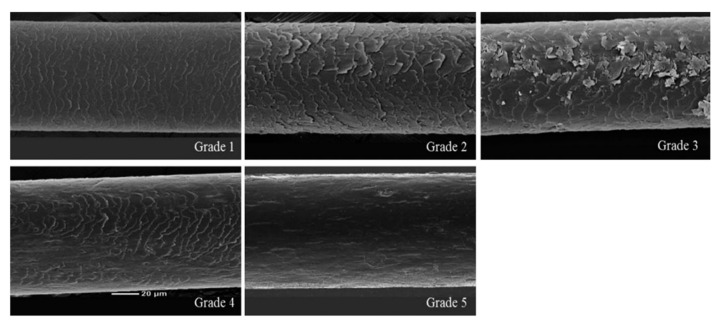
SEM standard photographs for grading criteria on a 5-point scale.

To assess the effect of CH ingestion on the shine of mouse hair, the hair surface was examined using a scanning electron microscope (FE-SEM-2, Sigma 300, Carl Zeiss, Germany). On day 21, five hairs were randomly plucked from each mouse. The experiment was conducted after acclimating the plucked hair at a temperature of 22 ± 1 °C and a relative humidity of 50 ± 5% for approximately 2 h. The hair samples were coated with gold using an automated sputter coater and then imaged using SEM. The SEM images were graded on a 5-point scale to evaluate the degree of damage to the hair surface and determine the shine of the hair (*n* = 8) (Table 3 and Figure 11).

### 4.7. Quantitative Reverse Transcriptase PCR for Cytokine Analysis

To determine the levels of cytokines involved in hair growth, total mRNA was extracted from the hDPCs and dorsal skin tissue of each C57BL/6 mouse, and cDNA was synthesized through reverse transcription. Real-time polymerase chain reaction was performed using a Piko-real 96 real-time PCR system (Thermo Fisher Scientific Inc., Waltham, MA, USA) with the following cycling conditions: 45 cycles at 95 °C for 15 s, 60 °C for 30 s, and 72 °C for 30 s. Table 4 provides the primer sequences used for amplifying IGF-1, VEGF, TGF-β1, TNF-α, IL-1β, Elastin, HAS2, and GAPDH.

### 4.8. Statistical Analysis

Statistical analyses were conducted using SPSS version 25 (SPSS Inc., Chicago, IL, USA). The data are presented as mean ± standard error (SE). The statistical analysis included a one-way analysis of variance (ANOVA), followed by Duncan’s multiple range test to determine mean differences. Statistical significance was defined as *p* < 0.05.

## Figures and Tables

**Table 1:** Effect of CH on hair length, weight, and density.

	Hair ^1^ Length (mm)	Hair Diameter (mm)	Hair Weight (g)	Hair Density (Score)
NC ^2^	15.95 ± 0.32 ^b 3^	0.36 ± 0.01 ^b^	0.019 ± 0.001 ^d^	48 ± 4.9 ^b^
PC	18.93 ± 0.44 ^a^	0.42 ± 0.01 ^a^	0.023 ± 0.001 ^c^	59.8 ± 3.8 ^a^
CH500	19.08 ± 0.41 ^a^	0.41 ± 0.01 ^a^	0.029 ± 0.001 ^b^	61.5 ± 3 ^a^
CH1000	19.95 ± 0.49 ^a^	0.43 ± 0.01 ^a^	0.035 ± 0.000 ^a^	68.8 ± 1.7 ^a^

^1^ Hairs were randomly plucked from the dorsal skin of mice on day 21. Hair diameter and length were measured using the ImageJ software (Java 8). Hair weight was determined by weighing half of the dorsal skin tissue sample with hair and subtracting the weight of the dorsal skin tissue sample from which the hair was removed. Hair density was measured using the DermoSmart+ Harris hair and scalp analyzer. ^2^ NC: negative control (distilled water); PC: positive control (finasteride); CH500: CH 500 mg/kg; CH1000: CH 1000 mg/kg. ^3^ Values with different superscript letters are significantly different (*p* < 0.05). The results are reported as the mean ± SE (*n* = 5/group).

**Table 2:** Scores for 4 grades of skin color.

Grade	Skin Color	Score
Grade 1	Pink (no hair)	0
Grade 2	Gray (initial hair growth)	1
Grade 3	Dark gray (visible hair growth)	2
Grade 4	Black (full-grown hair)	3

**Table 3:** Criteria for describing hair surface cuticle conditions on a 5-point scale.

Grade (Point)	Criterion
Grade 1 (1)	Intact hair (undamaged hair)
Grade 2 (2)	Irregular overlay, visible and lifted
Grade 3 (3)	Severely lifted and peeled
Grade 4 (4)	Cortex partially exposed
Grade 5 (5)	Cortex exposed without cuticle layer

**Table 4:** Primer pairs used for quantitative reverse transcriptase PCR.

Target Genes	Primer	Sequence
IGF-1	Forward	5′-TGCTCTTCAGTTCGTGTG-3′
	Reverse	5′-ACATCTCCAGTCTCCTCAG-3′
VEGF	Forward	5′-TCTTCAAGCCATCCTGTGTG-3′
	Reverse	5′-GCGAGTCTGTGTTTTTGCAG-3′
TGF-β1	Forward	5′-GGCGGTGCTCGCTTTGTAC-3′
	Reverse	5′-TCCCGAATGTCTGACGTATTGA-3′
TNF-α	Forward	5′-GCTGCACTTTGGAGTGATCG-3′
	Reverse	5′-TCACTCGGGGTTCGAGAAGA-3′
IL-1β	Forward	5′-CTTCTGGGAAACTCACGGCA-3′
	Reverse	5′-GTGAGACTCCAGACCTACGC-3′
Elastin	Forward	5′-TGGTGACATGATCCCTCTCTCTT-3′
	Reverse	5′-CCAGGGTGTCCCAGATGTG-3′
HAS2	Forward	5′-TGGCTGTGTCCAGTGCATAAG-3′
	Reverse	5′-CACAAATTCATGCAGCAAGGA-3′
GAPDH	Forward	5′-GGGAAGCCCATCACCATCT-3′
	Reverse	5′-CGGCCTCACCCCATTTG-3′

## Data Availability

The datasets used and/or analyzed during the current study are available from the corresponding author upon reasonable request.
